# Feasibility of Early Assessment of Cognitive Deficits in Patients With Ventilation Sepsis: A Cross-Sectional Study

**DOI:** 10.1016/j.arrct.2025.100547

**Published:** 2025-11-12

**Authors:** Frederick V. Ahlhaus, Marcus Vollmer, Michael Schröder, Matthias Gründling, Anke Steinmetz

**Affiliations:** a Physical and Rehabilitation Medicine, Department of Orthopaedics, Trauma and Rehabilitation Medicine, University Medicine Greifswald, Greifswald.; b Institute of Bioinformatics, University Medicine Greifswald, Greifswald.; c Department of Anaesthesiology and Intensive Care Medicine, University Medicine Greifswald, Greifswald.

**Keywords:** Cognition, Computer-assisted diagnosis, Critical care, Rehabilitation, Sepsis

## Abstract

**Objective:**

To evaluate the feasibility of early computer-based assessment, quantify cognitive impairments and identify factors influencing cognition.

**Design:**

Prospective, cross-sectional study.

**Setting:**

Data were collected on a surgical intensive care unit. All patients underwent cognitive assessment once they reached a Richmond Agitation-Sedation Scale (RASS) score of −1 or higher.

**Participants:**

During data collection, 60 patients (N=60) met the inclusion criteria: sepsis and 24 hours of ventilation. Patients with prior cognitive impairment, or neurologic or psychiatric diagnoses, were excluded. Therefore, a total of 28 patients were included in the study.

**Interventions:**

Not applicable.

**Main Outcome Measures:**

Primary outcome variables were tonic alertness, the general level of attention and phasic alertness, the temporarily increased attentiveness after a stimulus. They were normalized for age and sex. The hypothesis proposed was that testing would be feasible starting from a RASS score of −1.

**Results:**

Testing was only possible when patients had a RASS of 0 and had regained orientation. On average, a successful test could be performed 6.6 (mean) days from RASS≥−1 or 4.7 (mean) days after the end of ventilation. On average, the results of tonic alertness were 3 standard deviations (95% CI, −3.8 to −2.2) worse. For phasic alertness, the mean score was −2.3 standard deviations (95% CI, −3.1 to −1.5).

There are significant typical markers for sepsis severity with moderate correlations between an increasing number of ventilation days [*r*=−0.38, 95% CI: −0.66 to −0.01], number of days required between the cessation of ventilation and testing [*r*=−0.40, 95% CI: −0.68 to −0.04], days with noradrenaline [*r*=−0.48, 95% CI: −0.73 to −0.13], and days from awakening to the day of testing [*r*=−0.5, 95% CI: −0.74 to −0.15] and worsened tonic cognition.

**Conclusions:**

Early cognitive impairments are common in sepsis patients. Although early rehabilitation might be beneficial within this early stage, the utilization of computer-based assessments, appear to be limited. Detailed analysis of cognition should be considered later in recovery process. Further approaches could use demonstrated times at which assessment is likely to be successful.

## Highlights

• Sepsis impairs cognitive function, particularly alertness.
• Prolonged drowsiness negatively affects cognitive outcome.
• Future approaches should commence testing not too early.

Each year, 47 to 50 million people worldwide develop sepsis, with approximately 11 million deaths reported in 2017.^1^ Among survivors, about 75 % experience long-term consequences,^1^ including cognitive deficits.^2^ The incidence of sepsis is increasing, whereas mortality rates are decreasing,^3^ resulting in more patients surviving sepsis. Within the first year after discharge after sepsis, 74.3% of individuals develop a new health-related problem, with 18.5 % having a new cognitive deficit.^4^ Cognitive impairments lead to reduced quality of life and increased health care costs.^5^

Considering cognitive impairments, both long-term and acute cognitive impairments have been reported.^6^ Delirium^7^ and duration of mechanical ventilation^8^ have been identified as negative prognostic factors. Although preventive strategies for delirium exist in the intensive care unit (ICU), to our knowledge, there are no early rehabilitative interventions available for cognitively impaired postsepsis patients. Early assessment would facilitate rehabilitative concepts. This approach has proven effective in other conditions such as stroke or traumatic brain injury.^9^

As early cognitive rehabilitation is recommended in addition to preventing cognitive deficits^6^, it is essential to evaluate cognitive impairment using standardized tools and to determine when cognitive training is feasible. Several studies have examined cognitive impairments in ICU survivors,^7^^,^^8^ but little is known about how these deficits manifest across different domains of cognition. However, the influence of sepsis on early cognitive outcomes, remains to be delineated.

Computer-based software can assess and train cognitive deficits. Publications on computer-based rehabilitation^10^ approaches are promising. Its effectiveness on postsepsis patients remains unknown, although it has been recommended for critically ill patients.^11^ Initial studies using computer-based rehabilitation suggest reduced cognitive impairments and improved quality of life.^12^

To date, no standards have been established for cognitive testing in sepsis patients in ICU. Presently, the Richmond Agitation-Sedation Scale (RASS) remains the unique daily assessment, and this primarily serves to indicate vigilance rather than cognitive abilities.

In summary, this study aimed to determine if a computer-based assessment for cognitive impairments is feasible in clinical routine. With this method, the cognition in sepsis patients was assessed and influencing factors were evaluated.

## Methods

### Study group

For this clinical cross-sectional study, the participants were recruited from a surgical ICU. Recruitment occurred between March 21, 2023, and January 4, 2024. Patients were at least 18 years old and met the criteria for sepsis according to the Sepsis-3-defintion.^13^ Further inclusion criteria were at least 24 hours of ventilation, RASS≥–1, and informed consent.

Exclusion criteria included preexisting conditions that could further impair cognition, such as delirium and psychiatric disorders other than depression, preexisting intellectual disability or dementia, known alcohol or drug abuse, and central nervous system diseases with permanent cognitive impairments such as tumors or stroke sequelae. Furthermore, patients had to be able to use the RehaCom device. This requires intact motor function and adequate visual and hearing ability. Patients still affected by strongly sedating medications such as opioids, as well as those with severely limited life expectancy were excluded. Patients did not participate in any other cognitive training programs.

### Setting

**Fig 1:**
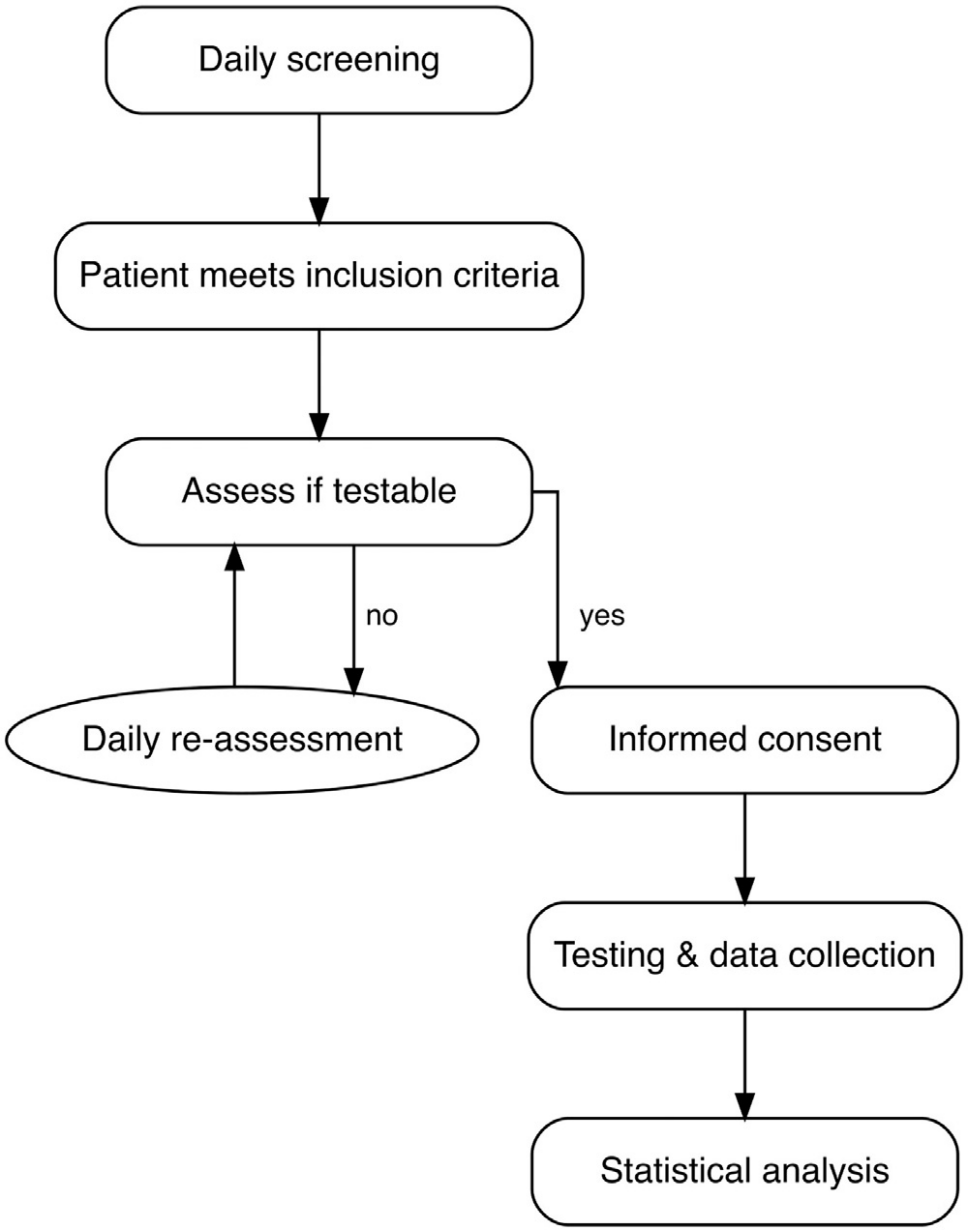
Curriculum of the study.

Once the patients met the inclusion criteria, daily attempts were made to conduct testing. For an overview of the study program, please refer to figure 1. Reasons for nonparticipation included severe drowsiness, delirium or insufficient compliance with instructions. It was frequently observed that patients either fell asleep during training sessions or did not pay sufficient attention, resulting in them simply clicking on random buttons. Before the test, potential distractions were minimized. If possible, the patient was placed in a more upright position. The RehaCom-patient keyboard was positioned on the patient’s lap near the dominant hand, and the tablet screen was in front of the patient. Reading glasses were provided if requested. During the assessments and tests, it was ensured that the patient could adequately perceive the acoustic stimuli presented during training. The volume was adjusted in consultation with the patient to optimize auditory clarity while preventing any discomfort or adverse effects. The module was explained, followed by the practice modules. Afterward, the testing was carried out. The test supervisor, trained in its administration, stood outside the subject’s field of view and gave no feedback during the measurement to avoid bias.

### RehaCom

The Alertness screening module measures the patient’s reaction speed and control. It primarily assesses their ability to respond quickly and adequately to a specific demand. This is a prerequisite for performing further cognitive-computer-based tests. First, as a simple reaction time measurement, the patient was instructed to press a button as quickly as possible when a square appeared. This assessed tonic alertness (AO0), the general level of wakefulness. For phasic alertness (AW0), the increased attention level, after stimulus, the device also emitted warning tones before the square appeared. The module consists of 4 blocks with the distribution A-B-B-A (A=without warning tone; B=with warning tone) to compensate for fatigue effects. The whole module lasts approximately 10 minutes, including practice, depending on the subject’s speed, and comprised at least 48 visual stimuli. If there was no response or the button was pressed incorrectly before the square appeared, stimuli reappear until 10 reactions are successfully registered.^14^ Completion of the ‘Alertness’ module was required for inclusion. The device is illustrated in supplemental figure S1 (available online only at http://www.archives-pmr.org/).

### Ethics

The Ethics Committee of University Medicine Greifswald approved the study protocol (BB 187/22, 28.03.2023). Reporting follows the STROBE guidelines. The study is registered in the German Clinical Trials Register (DRKS00031487).

### Data collecting

For statistical analysis, the z scores given by the RehaCom software were used for the dependent variables. These scores represented the patients’ cognitive outcomes, normalized for age and sex against a volunteer cohort provided by Hasomed, measured in standard deviations (SDs). These range from −5 (maximum impairment) to +2. A negative deviation of <1 SD or better is considered normal. A value between −1 and −2 SDs is abnormal but not necessarily pathologic. A result negatively deviating by 2 SDs from the mean is considered as severely impaired.^15^ The z-values in this context are equivalent to the SD of a Gaussian distribution curve. Consequently, a test subject with a z-value of +2 is positioned within the top 2.3% of the cohort provided by RehaCom.

Moreover, the patient’s medical history was reviewed to identify preexisting conditions that could potentially affect cognitive function. We looked for: cardiovascular diseases, diabetes mellitus, depression, chronic obstructive pulmonary disease, chronic kidney disease, rheumatoid arthritis, sleep disorders, malignant tumors outside the central nervous system, gastric ulcer, gout, visual impairment, hearing loss, neurologic disorders, except tumor, dementia, and stroke; the latter would have significantly restricted cognition before sepsis, which could falsify the data.

A range of conventional sepsis markers and demographic information were also documented as part of standard ICU data; ventilation days, type of ventilation (invasive or not), days without ventilation until testing (DNV), maximum required dose of noradrenaline, days with noradrenaline (DNA), and lactate levels at the time of sepsis were recorded. The severity of sepsis (sepsis/septic shock) and the Sequential Organ Failure Assessment (SOFA) score were determined on the day of sepsis diagnosis, as well as on the day of screening, and the difference between these values was calculated. It was identified whether cardiopulmonary resuscitation was necessary during the stay and whether the patients have received an operation or not. The number of days the patient met the inclusion criteria (RASS≥−1) until successful testing was calculated. Additionally, a questionnaire for verbal orientation from the Burgauer Bedside Screening (see supplemental appendix S1, available online only at http://www.archives-pmr.org/) was administered.

### Data analysis

Potential confounding factors, such as age, the severity of sepsis, and preexisting conditions, that could affect cognition, were identified. The effective control of confounding factors was rendered unfeasible by virtue of the inadequate sample size (n=28). Chi-square tests were conducted to evaluate biases in the cohorts to control for significant differences in preexisting diseases between groups.

Descriptive statistics, including mean, medians, 95% CI, SDs, minimum and maximum for continuous variables, as well as percentages for categorical variables, were calculated for baseline demographics and clinical characteristics.

Pearson correlations were used to analyze the dependence between AO0 and AW0 z scores, including 95% CI and *P* value for continuous variables. Correlation coefficients were interpreted as low (*r*<0.1), moderate (*r*<0.3), and high (*r*≥0.3) according to general guidance.^16^ To aid the interpretation of the clinical relevance of the observed effects, we also exploratively determined the effect size between the 2 groups, cognitive limited patients (z<−2) and not limited, using *t* tests. We calculated 95% CI for the effect size hedge`s g. Two-sided tests were applied with a preset alpha level of 0.05.

As one of the first studies to use RehaCom in critically ill patients, an initial power analysis was not feasible. The study population size was determined on the basis of a convenience sample, consistent with existing literature.

Data handling and statics ware performed with R^17^^,b^ in RStudio^18^^,c^ using the packages ggstatsplot,^19^ gtsummary,^20^ tidyverse,^21^ and dplyr.^22^ The data associated with the paper are not publicly available but are available from the corresponding author on reasonable request.

## Results

### Study population and demographics

**Fig 2:**
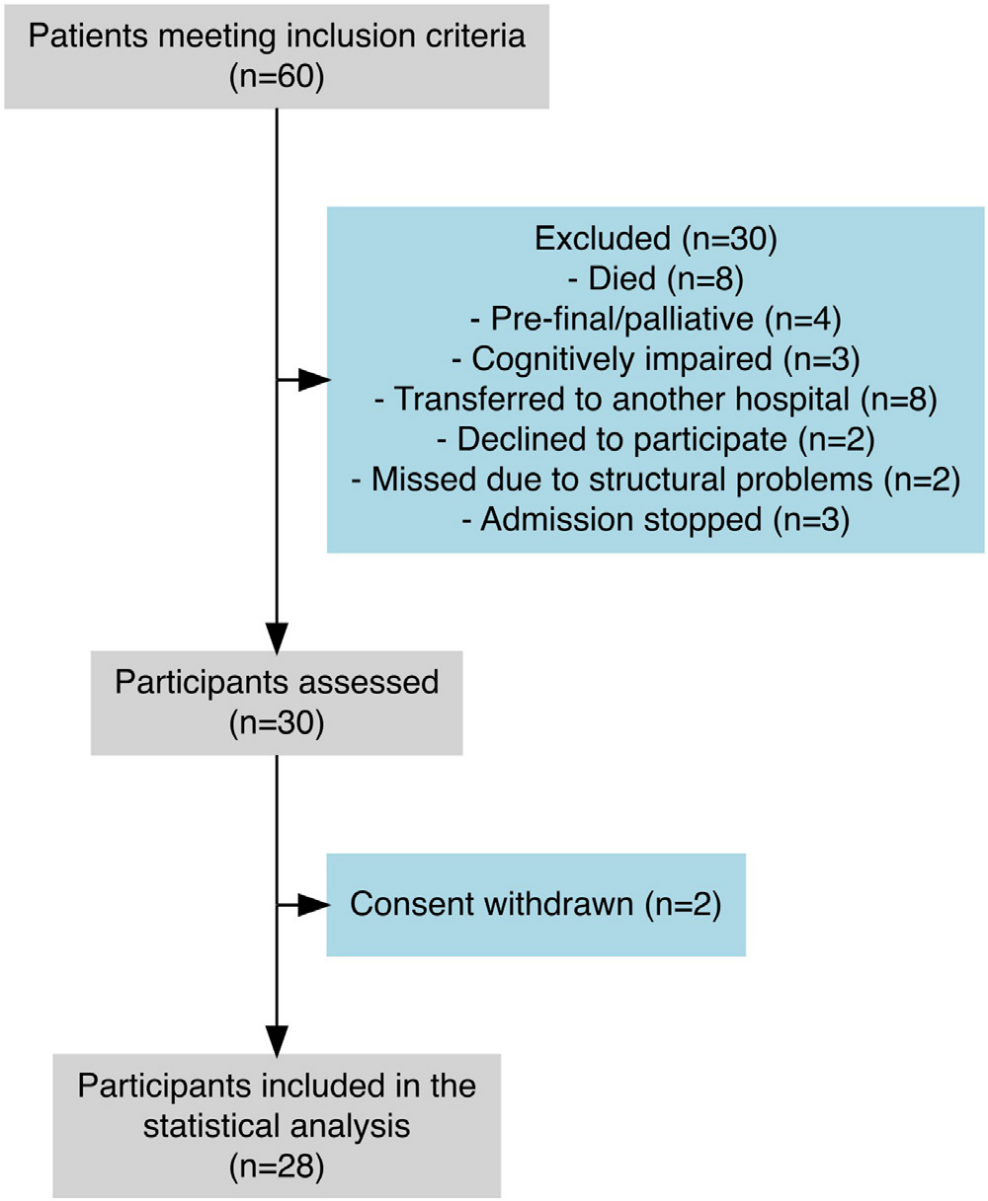
Flow diagram of inclusion, exclusion and cognitive assessment.

Sixty patients met the inclusion criteria. Data from a total of 28 participants are available for analysis. See flowchart (fig 2). The average age of our study population was 66 (minimum 41; maximum 89) years. Nine of the 28 patients were women. On average, patients were mechanically ventilated for 9 days. Overall, patients had 3 preexisting conditions. Eighty-nine percent suffered severe sepsis. Further values are provided in table 1. The distribution of preexisting conditions can be found in supplemental table S1 (available online only at http://www.archives-pmr.org/).

**Table 1:** Distribution of sex, age, preexisting conditions, treatment, and illness details of the participants.

Study Population Data	
Characteristic (unit)	Summary ± SD (MIN, MAX) or n (%)
Age at admission (y)	66.29±12.60 (41, 89)
Female sex	9 (32%)
Severity of sepsis (septic shock)	25 (89%)
Postoperative	27 (96%)
SOFA at time of sepsis	11.18±2.71 (7, 18)
Ventilation duration (d)	9.11±11.81 (1, 60)
Maximum dose of noradrenaline [mg/kg/min]	0.32±0.27 (0.02, 1.07)
Lactate at time of sepsis [mmol/l]	2.67±2.40 (0.40, 9.40)
Noradrenaline (d)	6.32±5.44 (1, 20)
Days RASS≥−1 to screening	6.64±5.52 (0, 23)
Orientation questionnaire (of 20 total points)	15.04±2.38 (8, 18)
SOFA day 0	2.93±2.28 (0, 8)
Difference SOFA sepsis - screening	8.25±3.06 (3, 16)
Days not ventilated: sepsis – screening	4.68±4.62 (0, 14)
RASS day 0 (=−1 / 0)	2 (7%) / 26 (93%)
Treatment days	33.96±20.00 (9, 91)

NOTE. Study population summary (n=28).

Abbreviations: CPR, cardiopulmonary resuscitation; ICU, intensive care unit; RASS, Richmond Agitation-Sedation Scale; SOFA, Sequential Organ Failure Assessment Score.

Overall, the subjects in this study tended to be severely affected by sepsis. Twenty-five of 28 had suffered septic shock. The average SOFA score was 11; organ damage can be assumed if the score is >2.^13^

### Criteria that facilitate the effective evaluation of cognitive function with RehaCom

Only 3 of the 28 patients could be assessed in more modules than alertness. At the time of testing the SOFA score was, on average, 3 (SD=2.28), with a maximum value of 8 and the SOFA score had improved by 8 points (SD=3.06) since the diagnosis of sepsis, with a minimum improvement of 3 points. The orientation questionnaire revealed that, on average, patients had returned to normal levels of orientation by the time of testing (mean= 15[of 20]; SD=2.38 [minimum=8; maximum=18]).

**Fig 3:**
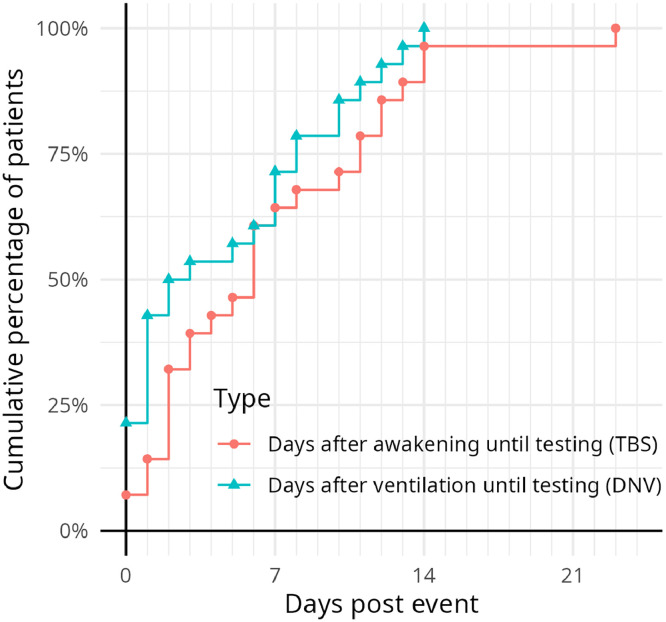
Cumulative number of patients included depending on day of awakening and days after ventilation until testing. (Richmond Agitation-Sedation Scale=−1). Abbreviations: DNV = days not ventilated; TBS = days from RASS ≥−1 to screening.

In addition, the duration of wakefulness before testing was assessed, defined as the number of days from when the RASS was ≥−1 until testing was possible. The hypothesis that testing could be conducted when the RASS reached −1 was not confirmed. In 26 of 28 patients (93%), testing was only possible once the RASS reached 0. The average number of days until the test is possible is 6.64 (SD=5.52), with a median of 6. For some patients, testing was possible immediately, whereas it took up to 28 days for others. However, by 14 days, nearly all patients (27/28=96%) could be tested (See fig 3).

Another parameter assessed was the number of days from the cessation of mechanical ventilation to the testing time. On average, 4.68 days (SD=4.62) passed before a successful assessment could be conducted. Six of 28 patients (21%) were testable while still on ventilation. Half of the patients were testable within 2 days of being off ventilation, and all patients could be tested no later than 14 days after stopping ventilation (See fig 3).

### Cognitive outcome

**Fig 4:**
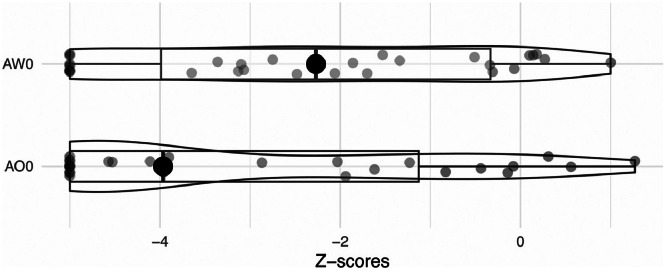
Boxplot of the cognitive outcome: tonic altertness (AO0) and phasic alertness (AW0). The big dot is the median value. The little dots show a single result for each participant and AO0 and AW0. Boxes showing quartiles.

For the general cognitive outcome (fig 4) for AO0, the mean z score was −3 (95% CI, −3.8 to −2.2), indicating severe mental impairment, with 10 patients reaching the maximum possible impairment (−5). The best score achieved was 1.27. For AW0, the mean z score was −2.31 (95% CI, −3.10 to −1.50) ranging from a minimum of −5 to a maximum of −1.5.

### Correlation of clinical parameters with cognitive outcome

Subsequently, we tested the linear correlation of individual continuous factors on the 2 dependent variables: AO0 and AW0. For AW0 the data suggest a high adverse correlation between noradrenalin days (DNA: *r*=−0.42; 95% CI=−0.69 to −0.06; *P*=.02) and treatment days (TD: *r*=−0.41; 95% CI = −0.68 to −0.04; *P*=.03) with the mental outcome. Age was highly positive correlated (*r*=0.46; 95% CI=0.11 to 0.71; *P*=.01).

Concerning AO0, an increasing amount of ventilation days (*r*=−0.38; 95% CI=−0.66 to −0.01; *P*=.05), number of days off ventilation until testing (DNV: *r*=−0.4; 95% CI=−0.68 to −0.04; *P*=.03), days with noradrenaline (DNA: *r*=−0.48; 95% CI=−0.73 to −0.13; *P*=.01) and days from awakening to the day of testing (TBS: *r*=−0.5; 95% CI=−0.74 to −0.15; *P*=.01) were highly correlated with worsened cognitive outcomes. There was a high positive correlation between higher scores on the orientation questionnaire and increased alertness (OQ: *P*=.39; 95% CI=0.02 to 0.67; *P*=.04). The corresponding correlation and *t* test values (see next paragraph) are provided in table 2 for AO0 and table 3 for AW0.

**Table 2:** Pearson correlation and *t* test for tonic alertness.

Correlation and *t* Test AO0			
	Correlation		*t* Test
Variable	Pearsons *r* (95% CI)	*P* Value	Hedges g (95% CI)
DNA	−0.48 (−0.73 to −0.13)	.01	−0.7 (−1.47 to 0.08)
TBS	−0.5 (0.74 to −0.15)	.01	−1.05 (−1.84 to −0.24)
DNV	−0.4 (−0.68 to −0.04)	.03	−0.79 (−1.56 to 0)
OQ	0.39 (0.02-0.67)	.04	1.12 (0.3-1.92)
VD	−0.38 (−0.66 to −0.01)	.05	−0.62 (−1.38 to 0.15)
TD	−0.37 (−0.65 to 0)	.05	−0.79 (−1.56 to 0)
S0	0.37 (−0.01 to 0.65)	.06	1 (0.2-1.79)
DS	−0.32 (−0.62 to 0.06)	.09	−0.75 (−1.52 to 0.03)
M0	0.31 (−0.08 to 0.61)	.12	0.53 (−0.25 to 1.3)
B0	−0.29 (−0.6 to 0.11)	.15	−0.52 (−1.3 to 0.27)
I0	0.23 (−0.16 to 0.55)	.25	0.43 (−0.33 to 1.19)
R0	0.17 (−0.22 to 0.51)	.40	0.41 (−0.35 to 1.17)
AGE	0.16 (−0.23 to 0.5)	.41	0.3 (−0.46 to 1.05)
PEI	0.15 (−0.23 to 0.5)	.44	0.26 (−0.49 to 1.01)
MNA	−0.1 (−0.45 to 0.29)	.62	−0.19 (−0.94 to 0.57)
STS	−0.06 (−0.42 to 0.32)	.77	−0.04 (−0.79 to 0.71)
LAC	−0.03 (−0.4 to 0.34)	.86	0.18 (−0.57 to 0.93)

NOTE. Pearson correlation of numeric variables and cognitive outcome. The results of the *t* test evaluate significant differences between groups, divided into cognitive limited and not for the variables.

Abbreviations: AGE, age at admission; AO0, tonic alertness; DNA, days with noradrenaline; DNV, days not ventilated: sepsis – screening; DS, SOFA score from sepsis until testing; LAC, lactate at time of sepsis ; TBS, days RASS≥−1 to screening; TD, treatment days; M0, medical research council sumscore (MRCss) day 0; B0, borg scale day 0; I0, IMS day 0; R0, RASS day 0; PEI, number of pre-existing conditions; STS, SOFA at time of sepsis; OQ, orientation questionnaire; VD, ventilation days; S0, SOFA day 0; MNA, maximum dose of noradrenaline.

**Table 3:** Pearson correlation and *t* test for phasic alertness.

Correlation and *t* Test AW0			
	Pearson Correlation		*t* Test
Variable	Pearson *r* (CI 95%)	*P* Value	Hedges g (CI 95%)
AGE	0.46 (0.11-0.71)	.01	1.08 (0.29-1.85)
DNA	−0.42 (−0.69 to −0.06)	.02	−0.73 (−1.48 to 0.02)
TD	−0.41 (−0.68 to −0.04)	.03	−0.72 (−1.46 to 0.03)
S0	0.37 (−0.01 to 0.65)	.05	0.7 (−0.05 to 1.44)
VD	−0.35 (−0.64 to 0.03)	.07	−0.6 (−1.33 to 0.14)
M0	0.34 (−0.05 to 0.64)	.08	0.35 (−0.39 to 1.08)
TBS	−0.33 (−0.62 to 0.05)	.09	−0.82 (−1.57 to −0.06)
OQ	0.33 (−0.05 to 0.63)	.09	0.38 (−0.35 to 1.11)
DS	−0.33 (−0.63 to 0.05)	.09	−1.17 (−1.95 to −0.37)
DNV	−0.29 (−0.6 to 0.09)	.13	−0.84 (−1.59 to −0.07)
B0	−0.25 (–0.58 to 0.14)	.20	−0.33 (−1.07 to 0.42)
PEI	0.25 (−0.14 to 0.57)	.20	0.57 (−0.17 to 1.3)
R0	−0.19 (–0.53 to 0.19)	.32	−0.59 (−1.32 to 0.16)
LAC	−0.09 (−0.44 to 0.3)	.67	0.04 (−0.68 to 0.76)
STS	−0.06 (−0.43 to 0.32)	.74	−0.6 (−1.33 to 0.14)
I0	0.06 (−0.32 to 0.43)	.75	−0.21 (–0.93 to 0.52)
MNA	0.02 (−0.35 to 0.39)	.91	−0.07 (−0.79 to 0.65)

NOTE. Pearson correlation of numeric variables and cognitive outcome. The results of the *t* test evaluate significant differences between groups, divided into cognitive limited and not for the variables.

Abbreviations: AGE, age at admission; AW0, phasic alertness; DNA, days with noradrenaline; DNV, days not ventilated: sepsis – screening; TBS, days RASS≥−1 to screening; TD, treatment days; M0, medical research council sumscore (MRCss) day 0; B0, borg scale day 0; I0, IMS day 0; R0, RASS day 0; PEI, number of pre-existing conditions; STS, SOFA at time of sepsis; OQ, orientation questionnaire; VD, ventilation days; S0, SOFA day 0; MNA, maximum dose of noradrenaline.

### Clinical parameter variations between cognitively impaired and nonimpaired participants

The participants of the study were divided into 2 groups based on their cognitive outcomes. This approach was further employed to ascertain the presence of any significant disparities in standard sepsis markers between the group afflicted with severe cognitive impairment and the group exhibiting no such impairment. The threshold was set at z=−2, representing the point where a patient is significantly impaired. Group A comprised participants with z scores ranging from −5 to −2 (inclusive), with n=18 for AO0. Group B included the remaining 10 participants with z scores above −2. A chi-square test (see supplemental tables S2 and S3, available online only at http://www.archives-pmr.org/) assessed the difference in binary variables like preexisting diseases between the groups. The results showed no significant difference in AO0. Only presence of gout showed a significant difference between the groups (chi-square: 3.88; *P*=.05) in AW0.

A *t* test was conducted for the numerical variables with the same group division. The results provide partial of the linear correlation analysis. The group with major impairment in AO0 was on average 1 SD later testable after awakening (TBS: hedges g=−1.05; 95% CI=−1.84 to −0.24; *P*=.01). The SOFA score at the time of testing (S0: hedges g=1.00; 95% CI=0.20 to 1.79; *P*=.03) and the results of the orientation questionnaire (OQ) were, on average, 1 SD higher in the group with better outcomes (OQ: hedges g=1.12; 95% CI=0.30 to 1.92; *P*=.00).

The group gaining a better cognitive outcome in AW0 had lower recovery in the SOFA score from sepsis until testing (hedges g=−1.17; 95% CI=−1.95 to −0.37; *P*=.00). On average, they could be tested about 1 standard deviation earlier after awakening (TBS: hedges g=−0.82; 95% CI=−1.57 to −0.06; *P*=.03) and after cessation of ventilation (DNV: hedges g=−0.84; 95% CI=−1.59 to −0.07; *P*=.03) than the cognitively impaired group. On average, they were about 1 standard deviation older (AGE: hedges g=1.08; 95% CI=0.29 to 1.85; *P*=.01).

## Discussion

The first studies using computer-based assessment in sepsis patients in the ICU employed a different test battery, more similar to the orientation questions.^23^ The observed correlation (AO0: *r*=0.39, *P*=.04) between the OQ’s results and those of the RehaCom tests is not unexpected. However, it remains unclear which assessment tool is best for obtaining a rapid cognitive overview^24^ remains unclear. Given the simplicity of the orientation questions, clinical staff can rapidly evaluate the cognitive status of patients. Further research is needed to explore the benefits in detail.

RehaCom is a powerful tool to assess all parts of cognition in more detail. Only 3 of the 28 participants could undergo further testing immediately afterward. Thus, only essential attentional capacity was assessed, leaving other cognitive domains untested.^15^

Early cognitive testing is generally challenging, as it requires substantial time and effort, with multiple attempts often necessary because of patients’ conditions. Based on our findings, testing should not be conducted before 14 days postawakening (RASS≥−1) or 14 days after the cessation of ventilation. It should also be noted that this early testing captures only immediate cognitive function and does not provide insights into long-term impairments, as seen in other studies.^2^

It is essential to investigate the extended postacute period further. Delirium in this period is an independent predictor of long-term cognition impairment.^7^ The cognitive outcome decreased with the days the patient was awake but not yet ready for testing (AO0: *r*=0.5, *P*=.01) The days without ventilation showed a similar trend (AO0: *r*=−0.4, *P*=.03)

This may suggest that implementing an early rehabilitative approach would improve the outcome. This is already seen in computerized cognitive rehabilitation with RehaCom, which enhances attention and executive function in acute and postacute brain injury patients.^25^ One limitation of our study is its insufficient sample size to determine which clinical parameters were specifically associated with prolonged “unalert” periods, during which patients could not be assessed. In the literature, very early physical and occupational therapy during interruptions of sedation results in better outcomes, such as a shorter duration of delirium.^26^ Early mobilization could improve long-term cognitive impairment,^27^ as it is already been demonstrated for physical function.^28^

Sepsis overall is known to have a severe impact on cognition.^2^ Because the syndrome cannot be directly measured, we used associated clinical parameters. No significant correlations were found between cognitive outcomes and some typical indicators of severe sepsis,^29^ such as lactate levels, maximum dose of noradrenaline, and the SOFA score at the onset of sepsis. Notably 89% of our patients were classified as having septic shock according to the Sepsis-3 criteria. This contrasts with the distribution of sepsis/septic shock in intensive care units, which is approximately 50%. Therefore, our cohort can be characterized as severely ill.^30^ This is also supported by high SOFA scores, and the requirement of at least 24 hours of ventilation. Overall, significant cognitive deficits were observed, suggesting a negative impact of sepsis on very early cognition, consistent with known impacts of sepsis on both early and long-term cognition.^2^ Patients who do not recover well from sepsis-related organ failure also have more cognitive problems. Conversely, cognitive abilities have been observed to improve with recovery, even in cases where the SOFA score remains elevated. This discrepancy could be explained by the hypothesis that these patients were tested at an earlier point in their disease. Other studies reported notable discrepancies between patients with and without infection.^31^ A potential approach to accurately assess the impact of sepsis on cognition may be to stratify by sepsis phenotypes. This is currently a topic of research with the aim of providing an explanation for the varying efficacy of treatments.^32^ The findings may facilitate the identification of candidates for early rehabilitative interventions. Revising the inclusion criteria to test and compare cases of milder sepsis without ventilation may be necessary for more detailed analyses. Additionally, comparing septic patients to an ICU control group with ventilation but without sepsis could help isolate the cognitive impact of sepsis itself. More detailed studies are essential to differentiate impairment because of sepsis from post-intensive care syndrome.^33^

### Study limitations

The generalizability of this study is limited because of single-center study design, the small study population recruited and a lack of heterogeneity. For instance, nearly all of the patients were postoperative and suffered severe sepsis, which could lead to a selection bias. Another area for improvement in this study may be that testing was conducted on weekdays. Variables such as days until testing was possible and days without ventilation until testing may have an inaccuracy of up to 2 days because there was no testing on weekends. Another limitation is that the effective control of confounding factors was rendered unfeasible by virtue of the inadequate sample size (n=28).

## Conclusions

Sepsis patients ventilated for at least 24 hours exhibited marked cognitive impairment, particularly in fundamental functions such as alertness. Sepsis-associated organ failure further compromised cognition. The longer patients were unable to participate in cognitive testing because of delirium or persistent drowsiness after regaining consciousness from the acute phase, the poorer their expected cognitive outcomes. Early assessments in the first days after awakening (RASS≥−1) are limited by inconsistent testability. Thus, the potential benefits of early ICU rehabilitation must be weighed against the challenge of optimal timing. At this stage, rehabilitation should be prioritized over quantification of impairment, whereas detailed cognitive assessment and targeted training appear to be more effective later in the recovery process. Future interventions should therefore be initiated after this point to improve testing efficiency.

## Suppliers

a. RehaCom; Hasomed.
b. R; R Core Team.
c. R Studio; Posit.

## Disclosure

Hasomed provided the RehaCom device for study proposes. M.G. regularly gives lectures for companies on the diagnosis and treatment of sepsis (Biomeriex, Sysmex, BD). The other authors have nothing to disclose.

## Appendix Supplementary materials

Image, application 1

Image, application 2

Image, application 3

Image, application 4

Image, application 5

## Footnotes

Supplementary material associated with this article can be found, in the online version, at doi:10.1016/j.arrct.2025.100547.
