# Supplementary figures and images for: Feasibility of Early Assessment of Cognitive Deficits in Patients With Ventilation Sepsis: A Cross-Sectional Study
Source: Arch Rehabil Res Clin Transl. 2025 Nov 12;8(1):100547. doi: 10.1016/j.arrct.2025.100547 (PMC12988563; doi:10.1016/j.arrct.2025.100547)

*Supplemental Figure S1: Rehabcom Device*


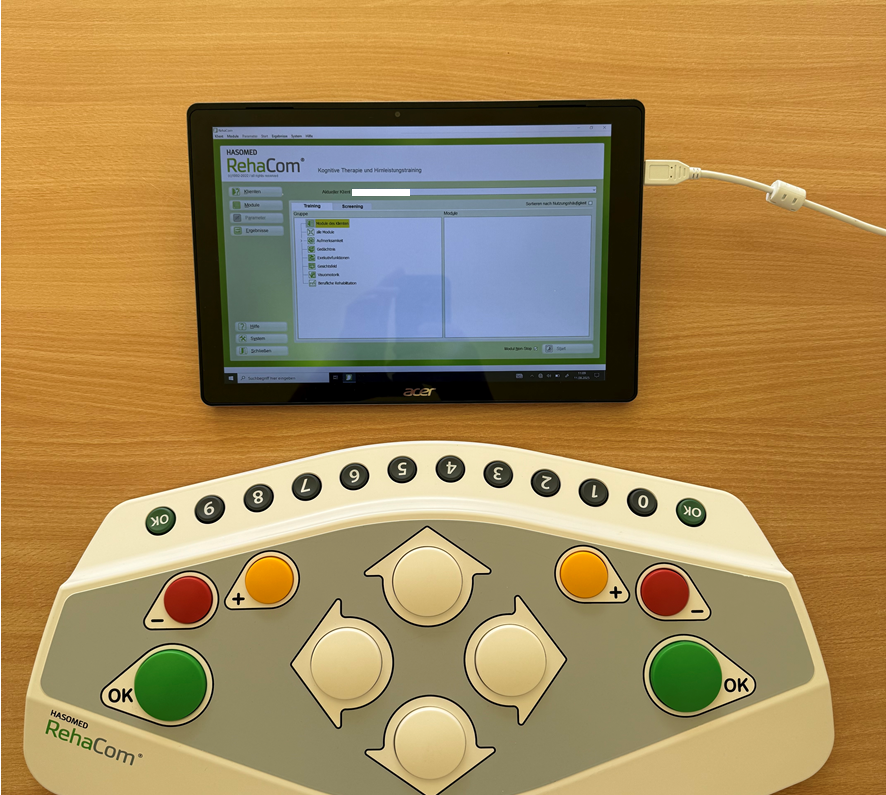

Supplement: Supplementary file 1 [file mmc1.docx]
