# Supplementary material for: Feasibility of Early Assessment of Cognitive Deficits in Patients With Ventilation Sepsis: A Cross-Sectional Study
Source: Arch Rehabil Res Clin Transl. 2025 Nov 12;8(1):100547. doi: 10.1016/j.arrct.2025.100547 (PMC12988563; doi:10.1016/j.arrct.2025.100547)
Supplement: Supplementary file 2 [file mmc2.docx]

**Supplementary Appendix**

ORIENTATION QUESTIONNAIRE

*Conducted in German. Here translated in English:*

***Personal Orientation:***

*What is your first name?*

*How old are you?*

*What is your profession?*

*What is your date of birth?*

*What is your current address? (Street and number)*

***Situational Orientation:***

*Why are you here? What happened?*

*What therapy are you receiving?*

*Who cares for you here at night?)?*

*Do you know someone by name here? (Staff)*

*What health insurance do you have?*

***Geographical Orientation:***

*In which city are you currently?*

*In which state are you currently?*

*What is the name of the hospital you are in?*

*On which floor/station are you right now?*

*In which direction would you have to travel from here to Berlin? (south)*

***Temporal Orientation:***

*What year is it?*

*What month is it?*

*What day of the week is it today?*

*What is today’s date? (Day, month, year)*

*Approximately what time is it right now? (± 1 hour)*
