# Supplementary material for: Feasibility of Early Assessment of Cognitive Deficits in Patients With Ventilation Sepsis: A Cross-Sectional Study
Source: Arch Rehabil Res Clin Transl. 2025 Nov 12;8(1):100547. doi: 10.1016/j.arrct.2025.100547 (PMC12988563; doi:10.1016/j.arrct.2025.100547)
Supplement: Supplementary file 3 [file mmc3.docx]

Supplementary Table S2

CHI-SQUARED-TEST AO0

| *Chi-squared-test AO0* | | |
| --- | --- | --- |
| *Variable* | *Chi-squared* | *p-value* |
| *GOU* | *3.32* | *0.07* |
| *IV* | *1.46* | *0.23* |
| *CKD* | *0.83* | *0.36* |
| *SD* | *0.83* | *0.36* |
| *CVD* | *0.12* | *0.73* |
| *SEX* | *0.00* | *1.00* |
| *DM* | *0.00* | *1.00* |
| *DEP* | *0.00* | *1.00* |
| *MT* | *0.00* | *1.00* |
| *ND* | *0.00* | *1.00* |
| *Supplementary table S2: Results of Chi-Quadrat-test with pre-existing conditios and tonic alertness (AO0). Groups divided by Z=-2. cardiovascular disease (CVD), diabetes mellitus (DM), depression (DEP), chronic obstructive pulmonary disease (COPD), chronic kidney disease (CKD), and rheumatoid arthritis (ART). sleep disorders (SD), malignant tumors (MT), gastric ulcer (GU), gout (GOU), visual impairment (VI), hearing loss (HL), and neurological disorders (ND)* | | |
