# Supplementary material for: Feasibility of Early Assessment of Cognitive Deficits in Patients With Ventilation Sepsis: A Cross-Sectional Study
Source: Arch Rehabil Res Clin Transl. 2025 Nov 12;8(1):100547. doi: 10.1016/j.arrct.2025.100547 (PMC12988563; doi:10.1016/j.arrct.2025.100547)
Supplement: Supplementary file 4 [file mmc4.docx]

Supplementary Table S1

PREEXISTING CONDITIONS

| **Preexisting conditions** | | **n ± SD [MIN, MAX] or n (%)** |
| --- | --- | --- |
| PEI | Number of pre-existing conditions | 3.11 ± 1.66 [0, 6] |
| CVD | Cardiovascular disease | 22 (79%) |
| DM | Diabetes mellitus | 7 (25%) |
| DEP | Depression | 6 (21%) |
| COPD | Chronic obstructive pulmonary disease | 4 (14%) |
| CKD | Chronic kidney disease | 7 (25%) |
| ART | Rheumatoid arthritis | 1 (3.6%) |
| SD | Sleep disorders | 7 (25%) |
| MT | Malignant tumors | 8 (29%) |
| GU | Gastric ulcer | 1 (3.6%) |
| GOU | Gout | 7 (25%) |
| VI | Visual impairment | 3 (11%) |
| HL | Hearing loss | 3 (11%) |
| ND | Neurological disorders | 11 (39%) |
| CPR | CPR during participation | 2 (7.1%) |
| PO | Post-operative | 27 (96%) |
| IV | invasive ventilation | 24 (86%) |
| Supplementary table S1: Distribution of preexisting conditions  SD= standard deviation, | | |
