# Supplementary material for: Feasibility of Early Assessment of Cognitive Deficits in Patients With Ventilation Sepsis: A Cross-Sectional Study
Source: Arch Rehabil Res Clin Transl. 2025 Nov 12;8(1):100547. doi: 10.1016/j.arrct.2025.100547 (PMC12988563; doi:10.1016/j.arrct.2025.100547)
Supplement: Supplementary file 5 [file mmc5.docx]

Supplementary Table S3

CHI-SQUARED-TEST AW0

| **Chi-squared-test AW0** | | |
| --- | --- | --- |
| **Variable** | **Chi-squared** | **p-value** |
| GOU | 3.88 | 0.05 |
| IV | 3.16 | 0.08 |
| CVD | 0.07 | 0.79 |
| DEP | 0.07 | 0.79 |
| DM | 0.05 | 0.83 |
| CKD | 0.05 | 0.83 |
| SD | 0.05 | 0.83 |
| SEX | 0.00 | 1.00 |
| MT | 0.00 | 1.00 |
| ND | 0.00 | 1.00 |
| *Supplementary table S3: Results of Chi-Quadrat-test with pre-existing conditions and phasic alertnesss (AW0). Groups divided by Z=-2. cardiovascular disease (CVD), diabetes mellitus (DM), depression (DEP), chronic obstructive pulmonary disease (COPD), chronic kidney disease (CKD), and rheumatoid arthritis (ART). sleep disorders (SD), malignant tumors (MT), gastric ulcer (GU), gout (GOU), visual impairment (VI), hearing loss (HL), and neurological disorders (ND)* | | |
